# Depressive Symptoms and Ageism among Nursing Home Residents: The Role of Social Support

**DOI:** 10.3390/ijerph191912105

**Published:** 2022-09-24

**Authors:** Dongjuan Xu, Yaqi Wang, Ming Li, Meng Zhao, Zhenhua Yang, Kefang Wang

**Affiliations:** 1 School of Nursing and Rehabilitation, Cheeloo College of Medicine, Shandong University, Jinan 250012, China; 2 School of Nursing, Purdue University, West Lafayette, IN 47907, USA

**Keywords:** ageism, depression, social support, nursing homes, modifying role

## Abstract

(1) Background: Ageism refers to the stereotyping, prejudice, and discrimination against older individuals or groups based on their age. This study investigates the modifying role of social support in the relationship between depressive symptoms and ageism in China; (2) Methods: A cross-sectional study was performed in 21 nursing homes in Jinan from March to June in 2019. The data were analyzed through a multilevel mixed-effects generalized linear model; (3) The analysis showed that older adults in nursing homes experienced moderate levels of ageism. There were significant interaction effects between depressive symptoms and social support on overall ageism and objective ageism after controlling for covariates (*p* < 0.05). As the level of social support increased, the predicted ageism greatly reduced among older adults without depressive symptoms when compared to those with depressive symptoms; (4) Conclusions: This study highlights the importance of identifying strategies to enhance social support and reduce depressive symptoms for nursing home residents. Having positive attitudes toward aging and overcoming negative age-related stereotypes may benefit older adults’ physical and mental health, well-being, and help to promote an age-friendly society.

## 1. Introduction

Ageism, a term that was first introduced in 1969 [1], refers to the stereotyping, prejudice, and discrimination against older individuals or groups based on their age. Ageism is more pervasive than racism and sexism [2], but it has not received the same level of attention nor is it widely countered. Previous research shows that ageism has negative consequences to the health and well-being of older adults [3]. A recent systematic review on ageism in 45 countries found a strong and consistent relationship between ageism and adverse health outcomes, including reduced longevity, cognitive impairment, physical and mental health, risky health behaviors, poor social relationships, and reduced quality of life [4]. Moreover, ageism is significantly related to reduced work opportunities and denied access to healthcare and treatments [4]. Therefore, it is particularly significant to identify and understand the motivation for ageism, which acts as a powerful barrier standing against an age-friendly society.

Perceived ageism varies significantly among countries. Around one-quarter of European older adults aged 62 years and over experienced discrimination due to their age, with 11% frequently experiencing it [5]. Almost half of Korean older adults aged 60–89 years experienced one or more incidences of ageism [6]. A recent study used identical measures from two nationally representative studies of aging and found that perceived age discrimination among adults aged over 52 years was significantly greater in England (38%) than the United States (29%) [7]. The differences may be explained by the different legislative environments (e.g., mandatory retirement age), cultural values, and social traditions (e.g., prejudicial attitudes toward age, negative stereotypes) across countries. 

While several studies have recently focused on ageism in western countries, there are far fewer studies in China. Investigating ageism among Chinese older adults is important for at least two reasons. First, decades of population control under the one-child policy, along with the mortality decline, increased the population aging proportion. Second, despite the Chinese tradition, which is based on Confucian ideals of respecting older people and viewing them as bearers of wisdom, values toward them are changing due to economic growth and modernization [8]. Various Chinese older adults view themselves as a burden to family and therefore perceive their lives to be less meaningful.

Among the factors influencing ageism, depressive symptoms play an important role. A recent study examined the bidirectional temporal relationship between depressive symptoms and perceived age discrimination in a cohort from the Health and Retirement Study, which included a representative sample of individuals over the age of 50 years in the U.S. These findings indicate that higher levels of depressive symptoms precede a greater likelihood of perceived age discrimination [9]. Another study found that even minor levels of depression were associated with a pattern of negative attitudes toward aging, and as depression intensity increased, negative attitudes were greater [10]. In contrast, social support has been identified as a protective factor, shielding older adults from the harmful effects of discriminatory experiences [11]. However, there has been little empirical research on the role of social support in the relationship between depressive symptoms and ageism. 

Ageism is a widespread phenomenon affecting health care [12], and it is more pronounced in long-term care than acute care [13]. Research on ageism in long-term care is scarce and most existing studies use qualitative methods [14]. To address this gap, we used a sample of nursing home residents in China to investigate (1) the level of perceived ageism by older adults and (2) the modifying role of social support in the relationship between depressive symptoms and ageism. Currently, more than 2.1 million Chinese older adults live in nursing homes [15]. Our findings may have important implications for the development of targeted interventions to combat ageism, improving health and well-being in long-term care facilities.

## 2. Materials and Methods

### 2.1. Participants and Procedure

This study was conducted from March to June 2019 in Jinan, the capital city of Shandong province, China. A purposive sampling method was used to recruit residents from 21 nursing homes. The selected nursing homes have operated for at least 1 year with 30 or more beds.

Inclusion criteria included: (1) being 60 years or older, which is used to define older people in China [16], (2) having lived in a nursing home for at least 3 months, and (3) willing to participate in the study. The exclusion criteria included: (1) having severe cognitive impairment, as indicated by the Mini-Mental State Examination (MMSE) total score <10 [17], (2) coma, end-stage disease, or receiving hospice care, and (3) severe vision and/or hearing problems, influencing survey completion. 

### 2.2. Measures

#### 2.2.1. Ageism

The Chinese Ageism Scale was used to assess nursing home residents’ feelings of ageism, which was developed based on the Fraboni Scale of Ageism [18]. The 22-item ageism questionnaire consisted of two subscales: subjective ageism and objective ageism, using a five-point Likert response scale (1 = strongly agree, 2 = agree, 3 = neutral, 4 = disagree, and 5 = strongly disagree). The average scores were calculated for overall, subjective, and objective ageism, respectively. A higher score indicated higher levels of ageism. The scale has been validated with Chinese older adults with good reliability and validity [19]. The Cronbach’s alpha was 0.81 in this study.

#### 2.2.2. Depressive Symptoms 

The Patient Health Questionnaire (PHQ-9) was used to assess nursing home residents’ depressive symptoms in the past 2 weeks [20]. Based on the frequency of feelings, questions are scored from 0 to 3 points (0 = none at all, 1 = several days, 2 = more than half the days, 3 = nearly every day). The total score ranges from 0 to 27. Individuals with a score of 5 or higher are likely to be suffering from mild or more severe depressive symptoms. Thus, depressive symptoms were used as a binary variable (above/below the 5-point cutoff). The scale has been validated for use in the nursing home population [21,22]. The Cronbach’s alpha was 0.79 in this study. 

#### 2.2.3. Social Support

The Lubben Social Network Scale (LSNS) was used to quantify social support perceived by nursing home residents [23]. The scale has 10 questions, each being assigned 0 to 5 points. The total score ranges from 0 to 50, with a higher score indicating more social support. The Cronbach’s alpha was 0.71 in this study. 

#### 2.2.4. Control Variables

Aside from the ageism, depressive symptoms, and social support, control variables included age, sex, marital status (married and divorced/widowed/never married), education level (illiterate, less than high school, high school or above), nursing home length of stay (less than 1 year, 1–3 years, and more than 3 years), cognitive impairment, activities of daily living (ADLs) dependency, number of chronic conditions, and anxiety symptoms. Cognitive impairment was assessed using the MMSE, and residents were grouped into none/mild or moderate [17,24]. The Barthel Index was used to assess ADLs dependency, and residents were categorized into independent and dependent groups [25]. The number of chronic conditions was a sum of 20, including hypertension, diabetes, angina, heart attack, congestive heart failure, stroke, arthritis, osteoporosis, prostate disease, asthma, chronic obstructive pulmonary disease and respiratory distress syndrome, or emphysema, nervous system disease, upper gastrointestinal disease, peripheral vascular disease, visual impairment, hearing damage, degenerative disc disease, kidney disease, cancer, and other diseases not mentioned above. The Generalized Anxiety Disorder Scale (GAD-2) was used to identify anxiety symptoms. Using the 3-point cutoff, residents were divided into two groups: having and not having anxiety symptoms [26]. As most residents (97.4%) were from the same ethnic group (Han), the ethnicity was not controlled in the analysis. 

### 2.3. Data Analysis

A descriptive analysis was used to describe the characteristics of nursing home residents, with mean and standard deviation for continuous variables and frequency and percentage for categorical variables. Ageism was our outcome variable. We conducted the Kolmogorov–Smirnova and Shapiro–Wilk tests for normality. Because the *p* values of both tests were greater than 0.05, the normality assumption of ageism was not violated. Residents were nested within nursing homes. To account for nested data, multilevel mixed-effects generalized linear models were conducted. The normal distribution (Gaussian) and an identity link function were used for model fitting, which had random intercepts for each nursing home. To investigate the modifying role of social support in the relationship between depressive symptoms and ageism, the interaction between depressive symptoms and social support was included in multivariate models. Multicollinearity was not an issue because the variance inflation factor (VIF) values of all variables were less than 10 [27]. All analyses were performed using Stata Version 16.1 (StataCorp, College Station, TX, USA). Statistical significance was set at the *p* < 0.05 (2-sided).

### 2.4. Data Collection

Researchers first contacted nursing home administrators and obtained their permission to investigate. Then, the trained research assistants approached residents in their facility and collected the data through face-to-face interviews. 

## 3. Results

**Figure 1:**
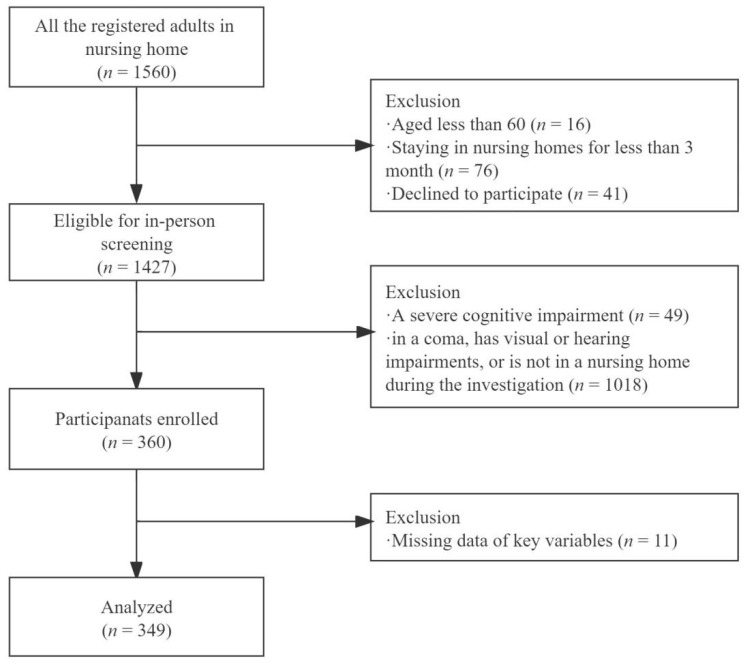
Study flow chart for sample selection.

Eligible residents (*n* = 360) from 21 nursing homes were enrolled in the study. Of these, 11 were excluded due to missing data. A flowchart of the study is shown in Figure 1. Table 1 describes participants’ characteristics. The average age was 77.9 years (range: 61–99 years). More than half (52%) were males and nearly 17% were married. Roughly 19% were illiterate, 54% did not graduate high school, and 28% had a high school or higher education. About 76% had lived in a nursing home for at least 1 year. Nearly 63% had no or mild cognitive impairment and 68% had ADL dependency. On average, residents had three chronic conditions. Approximately 9% had anxiety symptoms and 33% had depressive symptoms. The average score of social support was 19.3 (range: 0–47), which was relatively low. The respective average scores of ageism in overall, subjective, and objective domains were 2.8 (range: 1–4.5), 3.0 (range: 1–5), and 2.7 (range: 1–4.3) respectively.

**Figure 2:**
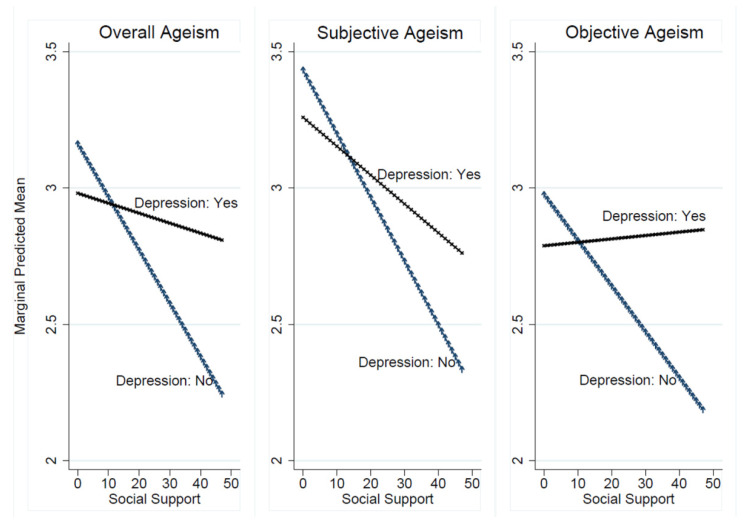
Relationship between depressive symptoms and ageism, the modifying role of social support.

As shown in Table 2, there were positive associations between depressive symptoms and ageism (overall: β = 0.136, *p* = 0.059; subjective: β = 0.092, *p* = 0.359; objective: β = 0.166, *p* = 0.016). Relative to those without depressive symptoms, residents with depressive symptoms experienced higher levels of ageism. As expected, there were negative associations between social support and ageism (overall: β = −0.013, *p* < 0.001; subjective: β = −0.016, *p* = 0.002; objective: β = −0.011, *p* = 0.002). As residents perceived higher levels of social support, they reported lower levels of ageism. Moreover, as shown in Table 3 and Figure 2, there were significant interaction effects between depressive symptoms and social support on overall ageism and objective ageism after controlling for sociodemographic and health-related variables. As the level of social support increased, the predicted ageism reduced to a greater extent among residents without depressive symptoms when compared to those with depressive symptoms (Figure 2). Additionally, among residents with depressive symptoms, social support was not related to objective ageism.

Besides depressive symptoms and social support, age had positive associations with ageism (overall: β = 0.010, *p* = 0.014; subjective: β = 0.014, *p* = 0.011; objective: β = 0.007 *p* = 0.076). As age increased, residents reported higher levels of ageism. Residents who obtained high school or higher level of education experienced less degree of subjective ageism compared with illiterate residents (β = −0.325, *p* = 0.043). Residents living in a nursing home for 3 years or more reported lower levels of subjective ageism than those living less than 3 years (3+ years vs. 1–3 years: β = −0.301, *p* = 0.005; 3+ years vs. <1 year: β = −0.341, *p* = 0.007). 

## 4. Discussion

Using a large sample of nursing home residents, this study provides evidence of the levels of ageism in the context of Chinese cultural values and social traditions. Consistent with previous studies [11,28], our results indicate that social support may play a protective role while depressive symptoms may play adverse roles relative to ageism. Moreover, as social support increased, the predicted levels of ageism reduced dramatically among residents without depressive symptoms when compared to those with depressive symptoms. 

In our study, nursing home residents experienced moderate levels of ageism. Ageing is usually accompanied by physical and mental decline, increased frailty, and causing loss of independence. These negative stereotypes, reflecting an intentional or unintentional devaluation of old age inherently, is one form of open and socially acceptable discrimination [29]. When compared to the findings of another study using the same measure of ageism among Chinese community-dwelling older adults [19], nursing home residents reported similar level of objective ageism and relatively higher level of subjective ageism. Older adults choose to live in nursing homes mainly because they do not want to be a burden to their children [30]. This view of themselves as a burden, held by various older adults, leads to a negative perception of themselves. 

Our findings support previous studies that indicated a positive association between depressive symptoms and perceived ageism [9,10]. People with depressive symptoms are more likely to view themselves, the world, and the future negatively [31]. This implies that depressed individuals may tend to not only view even neutral events as discriminatory, but also have negative emotional reactions to perceived ageism. Our results also suggest one-third of residents reported depressive symptoms, which is consistent with prior research [28,32]. Because of the high prevalence of depressive symptoms, it is crucial to detect and treat them early and routinely in long-term care settings.

Numerous studies have reported the importance of social support in maintaining physical and mental health and well-being, in which social support may act as a protective factor [33]. As expected, our finding suggests that higher levels of social support are significantly associated with lower levels of ageism after controlling for confounding variables, particularly among residents without depressive symptoms. Ageism appears to be a psychosocial stressor [3], and social support may confer resilience to stress [34]. Research demonstrated that intergenerational contact via family relationships and friendships may reduce negative attitudes toward age and age-related stereotypes [35]. In some Chinese traditions, the idea that one should raise children to look after them when they are old is highly accepted [36,37], and older adults have high expectations of support from family members in times of need, particularly from their offspring. Taken together, the findings underscore the significance of providing social support for nursing home residents, to effectively prevent or tackle ageism in long-term care settings.

This study found that age, education level, and length of stay were significantly associated with ageism. It was already expected that people would be more likely to experience ageism as they age [18]. As for education, residents with high school or above were less likely to perceive ageism, particularly subjective ageism, which aligned with a previous study’s results. Moreover, less educated older adults had a greater chance of experiencing the adverse health effects of ageism [4]. In addition, residents living in a nursing home for three years or more tended to perceive less ageism, particularly subjective ageism, which may reflect their positive adjustment to nursing care institutions. It is also possible that residents experienced increased dependence as they lived longer and increasingly internalized and submitted to ageist attitudes. 

### Limitations

There are several limitations to this study. First, the cross-sectional design does not allow us to conclude over the direction of causality between depressive symptoms, social support, and perceived ageism. Thus, we cannot exclude the reverse association. It could be possible that because nursing home residents experience less ageism, they report higher level of social support and lower levels of depressive symptoms. Second, all nursing homes located in urban areas and residents with severe cognitive impairment were not enrolled. Thus, the generalizability of these findings is limited. Third, although multilevel mixed-effects generalized linear models were conducted to account for residents clustered in nursing homes, facility characteristics such as leadership, organizational culture, and staff skill mix, which may have influences on residents’ perceived ageism, were not explored. Due to the limited knowledge of ageism among Chinese nursing home residents, our findings may serve as a starting point for more extensive and rigorous studies using prospective or experimental designs.

## 5. Conclusions

This study surveyed a large sample of nursing home residents and found that residents experienced moderate levels of ageism. This study highlighted the importance of identifying ways to enhance social support and reduce depressive symptoms for nursing home residents. Chinese older adults’ self-image and views of aging are often tied to their role in the family and to their relationships with family members. Efforts should focus on strategies or interventions to build, strengthen, and maintain family support as well as social support networks. Nursing facilities may prioritize the issue of ageism and take strategies to combat it, such as promoting social interaction and engaging residents to participate in physical activities. Nursing facilities should also raise staff awareness of ageism and work closely with them to build an equitable environment, such as proactive communication and training. Having positive attitudes toward aging and overcoming negative age-related stereotypes may not only benefit older adults’ physical and mental health and well-being, but also help to promote an age-friendly society.

## Figures and Tables

**Table 1:** Sample characteristics (*n* = 349).

	Mean ± SD or *n* (%)
Age (years)	77.93 ± 8.73
Sex	
female	167 (47.9%)
male	182 (52.1%)
Race/ethnicity	
Han	340 (97.4%)
other	9 (2.6%)
Marital status	
married	59 (16.9%)
divorced/widowed/never married	290 (83.1%)
Education level	
illiterate	65 (18.6%)
<high school	187 (53.6%)
≥high school	97 (27.8%)
Length of stay	
<1 year	84 (24.1%)
1–3 years	156 (44.7%)
>3 years	109 (31.2%)
Cognitive impairment	
no/mild	219 (62.8%)
moderate	130 (37.2%)
ADLs dependency	
independent	112 (32.1%)
dependent	237 (67.9%)
Number of chronic conditions	3.16 ± 2.27
Anxiety symptoms	
yes	31 (8.9%)
no	318 (91.1%)
Depressive symptoms	
yes	116 (33.2%)
no	233 (66.8%)
Social support	19.32 ± 9.02
Overall ageism	2.83 ± 0.64
subjective ageism	3.01 ± 0.88
objective ageism	2.70 ± 0.61

**Table 2:** Bivariate associations with ageism.

Construct	Overall Ageism			Subjective Ageism			Objective Ageism		
	β	*p*	95% CI	β	*p*	95% CI	β	*p*	95% CI
Depressive symptoms	0.136	0.059	−0.005,0.277	0.092	0.359	−0.104,0.288	0.166	0.016	0.031,0.302
Social support	−0.013	<0.001	−0.021,−0.006	−0.016	0.002	−0.026,−0.006	−0.011	0.002	−0.018,−0.004

Note: β: regression coefficient; CI: confidence internal; Boldface indicates statistical significance (*p* < 0.05).

**Table 3:** The modifying role of social support in the relationship between depressive symptoms and ageism.

Construct	Overall Ageism			Subjective Ageism			Objective Ageism		
	β	*p*	95% CI	β	*p*	95% CI	β	*p*	95% CI
Depressive symptoms	−0.211	0.202	−0.534,0.113	−0.187	0.412	−0.635,0.260	−0.228	0.156	−0.543,0.087
Social support	**−0.020**	**<0.001**	−0.029,−0.010	**−0.023**	**0.001**	−0.037,−0.010	**−0.017**	**<0.001**	−0.026,−0.008
depression * social support	**0.016**	**0.044**	−0.001,0.031	0.013	0.241	−0.009,0.034	**0.018**	**0.019**	0.003,0.033
Age	**0.010**	**0.014**	0.002,0.018	**0.014**	**0.011**	0.003,0.025	0.007	0.076	−0.001,0.015
Sex (ref. male)									
female	0.010	0.891	−0.139,0.159	0.058	0.582	−0.149,0.265	−0.024	0.742	−0.167,0.119
Marital status (ref. unmarried)									
married	−0.017	0.851	−0.195,0.160	−0.064	0.612	−0.309,0.182	0.016	0.853	−0.156,0.189
Education level (ref. illiterate)									
<high school	−0.096	0.333	−0.289,0.098	−0.132	0.338	−0.401,0.137	−0.068	0.478	−0.256,0.120
≥high school	−0.197	0.087	−0.422,0.029	**−0.325**	**0.043**	−0.639,−0.011	−0.106	0.339	−0.323,0.111
Length of stay (ref. < 1 year)									
1–3 years	−0.012	0.887	−0.177,0.153	−0.040	0.733	−0.267,0.188	0.011	0.896	−0.149,0.171
>3 years	−0.163	0.072	−0.341,0.015	**−0.341**	**0.007**	−0.588,−0.093	−0.039	0.651	−0.210,0.131
Cognitive impairment (ref. no/mild)									
moderate	−0.067	0.392	−0.221,0.087	−0.086	0.426	−0.299,0.126	−0.052	0.493	−0.203,0.098
ADLs dependency(ref. independent)									
dependent	0.035	0.627	−0.108,0.178	−0.059	0.557	−0.258,0.139	0.103	0.145	−0.035,0.241
Number of chronic conditions	0.003	0.851	−0.028,0.034	0.019	0.392	−0.024,0.061	−0.008	0.590	−0.038,0.022
Anxiety symptoms	0.128	0.302	−0.115,0.371	0.106	0.536	−0.231,0.443	0.142	0.236	−0.093,0.378

Note: ADLs: activities of daily living; β: regression coefficient; CI: confidence internal; Boldface indicates statistical significance (*p* < 0.05).

## Data Availability

The datasets are not publicly available during the current study, but data are available from the applicants upon reasonable request and with permission of the researcher’s University.
